# The Responsibility-Sharing of Nation-States and the ACT-Accelerator

**DOI:** 10.34172/ijhpm.2021.40

**Published:** 2021-05-05

**Authors:** Minju Jung, Simon Rushton

**Affiliations:** Department of Politics and International Relations, University of Sheffield, Sheffield, UK.

## Introduction

To bring the coronavirus disease 2019 (COVID-19) pandemic to an end and begin the process of social, economic and public health recovery, international cooperation for the continued development and equitable distribution of high-quality diagnostics, therapeutics and vaccines is necessary. Although critics have rightly pointed out that achieving universal coverage and equitable access to these health technologies would require reforms to intellectual property rules as well as increased global manufacturing capacity,^1^ in the meantime the ‘Access to COVID-19 Tools Accelerator’ (ACT-Accelerator), led by the World Health Organization (WHO), is the only global multilateral effort designed to ensure the worldwide distribution of these products. In this viewpoint, we focus on what it would take to fully fund this mechanism and make it successful on its own terms.

The ACT-Accelerator was launched by the WHO and its partners (including France, the European Commission, and the Bill & Melinda Gates Foundation) one year ago, on April 24, 2020. The framework aims to facilitate the development and production of COVID-19 diagnostics, therapeutics and vaccines, and to ensure affordable and equitable access to these resources globally.^2^ To achieve these goals, it developed four pillars: Vaccines, Therapeutics, Diagnostics, and the ‘Health Systems Connector’ pillar.^3^ The Vaccine pillar (COVAX), which has been by far the most high-profile element of the ACT-Accelerator, aims to provide 2 billion vaccine doses globally by the end of 2021. The Therapeutics pillar aims to provide 245 million therapeutics for low and middle-income countries by mid-2021, and the Diagnostics pillar aims to test 500 million cases in low and middle-income countries by mid-2021. The Health Systems Connector pillar aims to support the other three pillars by improving health systems and local community networks in developing countries.

As of September 2020, the ACT-Accelerator framework was estimated by the WHO to require a total of US$38 billion to achieve its goals across the four pillars.^3^ However, global fundraising efforts to support the ACT-Accelerator had only raised US$16.9 billion by March 12, 2021, resulting in a significant gap in funds. In this viewpoint, we argue that (especially given the inability to agree any meaningful changes to the prevailing intellectual property rules), WHO member states have a moral and ethical responsibility to at least ensure the ACT-Accelerator is fully funded. We argue that, so far, the responsibility to mobilise the necessary financial resources has been shared disproportionately, with middle-income countries in particular having not shouldered their share of the burden.

## The Human Right to Health and the Responsibility of States to Fully Fund the ACT-Accelerator

Human rights are “*rights inherent to all human beings, regardless of race, sex, nationality, ethnicity, language, religion, or any other status*.”^4^ According to the WHO’s Constitution, “*The enjoyment of the highest attainable standard of health is one of the fundamental rights of every human being without distinction of race, religion, political belief, economic or social condition*.”^5^

The International Covenant on Economic, Social and Cultural Rights, adopted in 1966, specified in Article 12.2 that one component of the right to health is ‘the right to prevention, treatment and control of diseases’^6^ (p. 8). In its ‘General Comment 14’ in 2000, the United Nations’ (UN’) Committee on Economic, Social and Cultural Rights (CESCR) sought to more clearly define the normative content of the right to health.^7^ In Paragraph 16, the CESCR focussed on the right to treatment and control of diseases. They noted that this right, inter alia, required governments to implement disease prevention and education programmes, to promote the social determinants of good health, and to ensure the availability of emergency medical care. Of most direct relevance for the current discussion, they stated that “*The control of diseases refers to States’ individual and joint efforts to, inter alia, make available relevant technologies…*.” According to the CESCR, therefore, the ACT-Accelerator’s support for the development and distribution of medical technologies, and the health system capacities to deliver them, is an essential part of realising the right to prevention, treatment and control of diseases (and beyond that, the right to health).

 Who has the responsibility for the realisation of these rights in practice? Nation-states have traditionally been understood as having responsibilities first and foremost to their own citizens. Yet General Comment 14 specifically sought to assign a collective responsibility on all states in the event of a pandemic:

“*Given that some diseases are easily transmissible beyond the frontiers of a State, the international community has a collective responsibility to address this problem. The economically developed States parties have a special responsibility and interest to assist the poorer developing States in this regard” *(Paragraph 40).

In addition to these Right to Health-based arguments, scholars in International Law (eg, Kavanagh et al^8^) have argued in the case of antiretroviral HIV medicines that equitable global access is also demanded by the human right to benefit from scientific advances. If so, there seems no reason to suppose that such a right does not equally apply to diagnostics, vaccines, and treatment for COVID-19. There seem, therefore, to be strong rights-based arguments for a collective responsibility to deliver on the four pillars of the ACT-Accelerator^[1]^.

Given that countries have differential capacities, however, there remains a question as to how this collective responsibility should be distributed among states (not least financially). As one possible way of allocating responsibility, we suggest a principle of proportionality under which states should take responsibility for funding the global realisation of the right to prevention, treatment, and control of COVID-19 through funding the ACT-Accelerator in proportion to the size of their economies – specifically their share of global gross national income (global GNI). This proportionality principle in global public goods provisions also exists in other regimes designed to manage global public goods coordination, such as in the UN Framework Convention on Climate Change.^9,10^

## Examining Nation-States’ Financial Contributions to the ACT-Accelerator

The idea that states have a responsibility to contribute to the ACT-Accelerator framework in proportion to their share of global GNI provides an illuminating way of benchmarking contributions so far. To investigate this, we analysed the financial resource commitments of 203 countries (eg, the territories recognized as nation-states by the World Bank) to the ACT-Accelerator up to March 12, 2021. The data were collected from the Economist Intelligence Unit’s *COVID-19 Health Funding Tracker*^[2]^.^11^ These were compared with countries’ relative wealth, as measured by the World Bank’s 2018 global GNI index^[3]^, which calculates countries’ GNI (in current US dollars) in 2018^[4]^. These countries were classified by the World Bank into four income groups: high-, upper middle-, lower middle- and low-income.^12^

Overall, the amount raised for the ACT-Accelerator by March 12, 2021 totalled US$16.91 billion – far less than the goal of US$38 billion. Non-state donors^[5]^ accounted for 31% of the total contributions so far; state donors account for 69% of funds raised (a total of US$11.67 billion). Out of 203 countries, 68 countries had committed funds as of 12 March.

When the relative contributions are examined according to the World Bank’s four income groups, it is revealed that there are significant differences. As Table 1 shows, no income group had pledged its ‘share’ of the total required to fully fund the ACT-Accelerator as of 12 March. Even the high-income group, which unsurprisingly accounts for the largest share of state donations by far (a total pledge of US$11.49Bn) has only pledged just over 55% of the amount that would constitute its full share of just over US$20Bn. Perhaps more surprisingly, the low-income group comes in second place, having pledged over 41% of its full share. So far, middle-income countries are the group that have failed to contribute in line with their share of global GNI by the largest margin. Upper middle-income countries account for 28.79% of global GNI but only 0.9% of contributions to the ACT-Accelerator, which amounts to only just over 1% of their share. Lower middle-income countries, meanwhile, make up 6.91% of global GNI but only 0.02% of contributions so far.

**Table 1:** Nation-States’ Contribution to the ACT-Accelerator by Global Income Group (as of March 12, 2021)

**Income Group** ^a^	**Number of Countries**		**Share of Global GNI in 2018** ^b^	**Expected Contribution (If Contributions Were Proportionate to GNI and the ACT-A Was Fully-Funded)**	**Actual Contribution** ^c^		**Actual Pledge vs. Expected Contribution **	
	**No. of Countries **	**No. of Contributing Countries in Group**	**%**	**US$ (Bn)**	**US$ (Bn)**	**%**	**% Of Expected Contribution Actually Pledged**	**‘Funding Gap’ Between Expected Contribution and Actual Pledge (US$(Bn))**
High-	**68**	**40**	**62.87**	**20.79**	**11.49**	98.49	55.29	-9.30
Upper middle-	**56**	**13**	**28.79**	**9.52**	**0.11**	0.90	1.11	-9.41
Lower middle-	**50**	**7**	**6.91**	**2.29**	**0.002**	0.02	0.11	-2.28
Low-	**29**	**8**	**0.50**	**0.17**	**0.07**	0.59	41.28	-0.10
Total	**203**	**68**	**99.07** ^d^	**32.76** ^e^	**11.67**	**100 %**	**35.62** ^f^	**-21.09**

Abbreviations: ACT-A, Access to COVID-19 Tools Accelerator; GNI, gross national income ^a^As per World Bank classification.^12^ ^b^The global GNI in 2018 (about US$ 86.3 trillion). ^c^The total funds that nation-states pledged as of March 12, 2021 (US$ 11.67 billion). ^d^This column does not total 100% as there are some territories that are not recognised by the World Bank as countries, but nevertheless account for a (small) share of Global GNI. ^e^US$ 32.76 billion = The amount of funds that WHO requested for the ACT-A (US$ 38 billion) minus the contribution of non-state actors (private sector, multilateral donors, philanthropists etc) (US$ 5.24 billion as of March 12, 2021). ^f^ie, only 35.62% of the US$32.76 Bn required to fully fund the ACT-Accelerator had been pledged by March 12, 2021.

Breaking these categories down reveals further disparities in contribution, and a further tranche of countries in the ‘missing middle:’ namely, the lower parts of the high-income group. Although the high-income group have been the biggest donors by far, the vast majority of these funds have come from the G7 (see Table 2) – and even these have accounted for only 64% of the G7’s ‘share’ according to their share of global GNI. The non-G20 members of the high-income group contributed only 10.85% of the total amount pledged so far, despite the fact that their GNIs accounted for 12.61% of the global GNI of 2018. Several G20 members had not pledged at all as of 12 March. The non-G7 countries of the G20, contributed even less: only 4.72% of contributions compared to their 32.1% share of the global GNI.

**Table 2:** High-income Countries’ Financial Resources Commitments (as of March 12, 2021)

**Income Group** ^a^	**Number of Countries**		**Share of Global GNI in 2018** ^b^	**Expected Contribution (If Contributions Were Proportionate to GNI and the ACT-A Was Fully-Funded)**	**Actual Contribution** ^c^		**Actual Pledge vs. Expected Contribution **	
	**No. of Countries **	**No. of Contributing Countries in Group**	**%**	**US$ (Bn)**	**US$ (Bn)**	**%**	**% Of Expected Contribution Actually Pledged**	**‘Funding Gap’ Between Expected Contribution and Actual Pledge (US$(Bn))**
High-	68	40	**62.87**	20.79	11.49	98.49	55.29	-9.30
The G7 group^d^	7	7	**45.74**	15.12	9.70	83.15	64.16	-5.42
The non-G7 countries of the G20^e^	12	7	**32.10**	10.61	0.55	4.72	5.19	-10.06
The non-G7 countries of the high-income countries	61	33	**17.13**	5.66	1.79	15.34	31.61	-3.87
The non-G20 countries of the high-income Countries^f^	58	30	**12.61**	4.17	1.27	10.85	30.37	-2.90

Abbreviations: ACT-A, Access to COVID-19 Tools Accelerator; GNI, gross national income; Bn, billion. ^a^As per World Bank classification.^12^ ^b^The global GNI in 2018 (about US$ 86.3 trillion). ^c^The total funds that nation-states pledged as of March 12, 2021 (US$ 11.67 billion). ^d^ US$ 32.76 billion = The amount of funds that WHO requested for the ACT-A (US$ 38 billion) minus the contribution of non-state actors (private sector, multilateral donors, philanthropists etc) (US$ 5.24 billion as of March 12, 2021). ^e^Canada, France, Germany, Italy, Japan, the United Kingdom and the United States. ^f^These 12 countries are Argentina, Australia, Brazil, China, India, Indonesia, Republic of Korea, Mexico, Russia, Saudi Arabia, South Africa, Turkey (we exclude the final G20 member: the EU).

## Conclusion

 Even fully funding the ACT-Accelerator may not be sufficient to ensure universal access to vital COVID-19 technologies – other interventions and reforms will likely be required. However, the shortfall in contributions so far dooms the framework to fail even on its own terms. In this commentary, we investigated the financial contribution of state donors to the ACT-Accelerator so far, in comparison to their economic capability measured in GNI in 2018. We found that first, the total amount of contributions is much less than the ACT-Accelerator requested (only 35% of the amount needed as of March 12, 2021). Second, most of the contributions came from a few of the world’s most economically developed countries (ie, the G7). Third, the contributions of non-G7 high-income countries and middle-income countries (both upper and lower) were much lower in comparison to their economic ability, suggesting that (in contrast to the low-income group) they have so far failed to shoulder their share of the burden.

## Acknowledgment

We would like to thank both the editor and reviewer of *International Journal of Health Policy and Management* for their valuable comments for this manuscript.

## Ethical issues

 Not applicable.

## Competing interests

 Authors declare that they have no competing interests.

## Authors’ contributions

 MJ designed this study and drafted the first version of this manuscript. SR provided critical review and revision of the manuscript. Both authors approved the final draft of the manuscript.

## Endnotes


[1] The idea that governments have a collective responsibility for the realisation of human rights is not limited to the Right to Health. Such a responsibility has also, for example, been argued for in the case of the rights of refugees. Dowd and McAdam,^13^ for example, argue that the protection and hosting of refugees should be a responsibility shared collectively among states, regardless of whether they contributed to the cause of the refugee flows in the first place [also see ^14^].

[2] The Economist Intelligence Unit’s COVID-19 Health Funding Tracker (https://covidfunding.eiu.com/) provides independent data on “global, health-related funding efforts, from pledge to disbursement.” The tracker divides funding into 10 funding streams. In this viewpoint, we utilised the pledge data (it is important to note that a significant number of pledges have not yet proceeded to disbursement) allocated to the six streams most directly related to the ACT-Accelerator, namely: ‘ACT-A to be confirmed,’ ‘ACT-Accelerator Vaccines,’ ‘ACT-Accelerator Therapeutics,’ ‘ACT-Accelerator Diagnostics,’ ‘ACT-Accelerator Health systems,’ and ‘WHO Strategic preparedness and response plan.’
 [3] In 2018, global GNI amounted to about US$ 86.3 trillion.
[4]For a few missing data, we utilised the UN’s 2018 GNI index.^15^
 [5] These non-state donors include multilateral organisations (eg, United Nations Children’s Fund), regional institutions (eg, the European Investment Bank), and private sector organizations (eg, philanthropic foundations, private companies and individuals).
